# Supplementary material for: Quantification of cell behaviors and computational modeling show that cell directional behaviors drive zebrafish pectoral fin morphogenesis
Source: Bioinformatics. 2021 Mar 24;37(18):2946–54. doi: 10.1093/bioinformatics/btab201 (PMC10262313; doi:10.1093/bioinformatics/btab201)
Supplement: btab201_supplementary_data [file btab201_supplementary_data.pdf]

Bioinformatics
doi.10.1093/bioinformatics/xxxxxx
Advance Access Publication Date: Day Month Year
Manuscript Category

Supplementary material for

Quantification of cell behaviors and computational modelling show that cell directional behaviors drive zebrafish pectoral fin morphogenesis

Joel Dokmegang [1,*], Hanh Nguyen [2], Elena Kardash [2], Thierry Savy [2,3], Matteo Cavaliere [1], Nadine Peyriéras [2,3,*] and René Doursat [2,3,*]

[1]Centre for Advanced Computational Science, Manchester Metropolitan University, Manchester, M15 6BH, United Kingdom,
[2]BioEmergences, FRE2039, CNRS Université Paris Saclay, Gif-sur-Yvette, 91190, France and
[3]Complex Systems Institute, Paris Ile-de-France, Paris, 75013, France.

picture(0,0)(-35,0)(1,0)30 (0,35)(0,-1)30 picture picture(0,0)(35,0)(-1,0)30 (0,35)(0,-1)30 picture

Supplementary figure 1

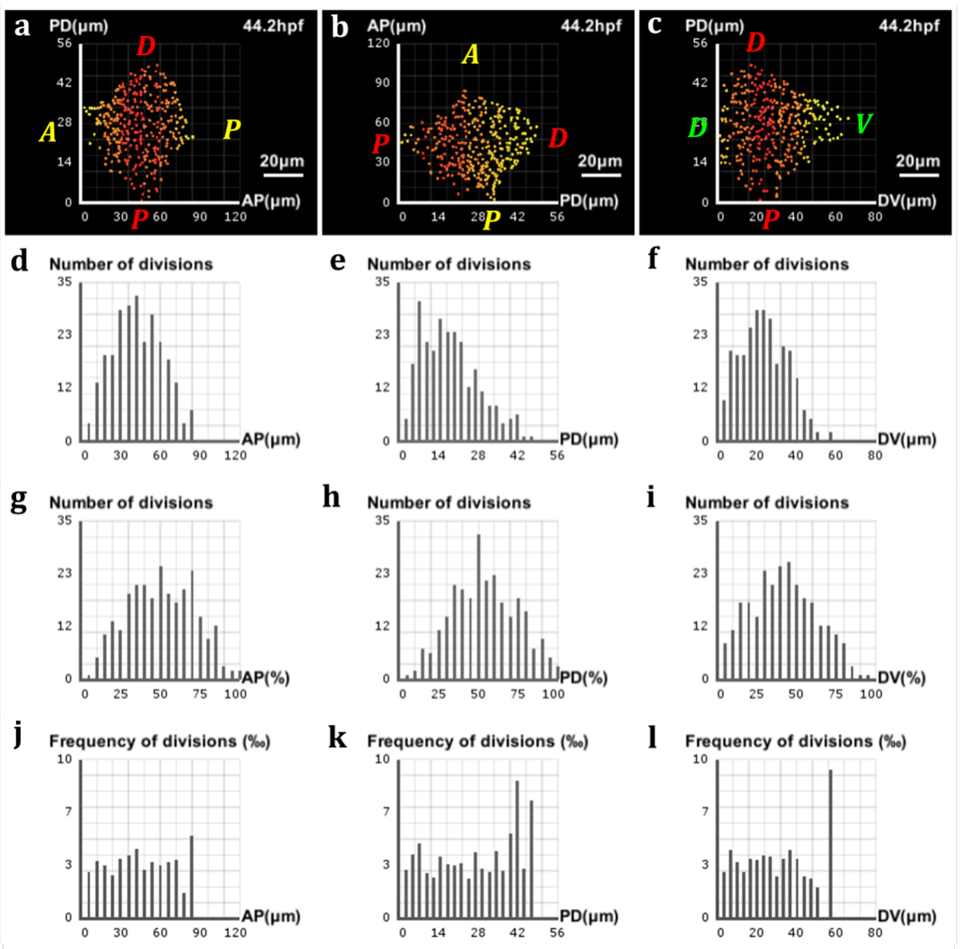

Fig. S1. Analysis of proliferation in the zebrafish pectoral fin (supplementary dataset). (a-c) Frequencies of divisions along the AP, PD and DV axes respectively, highlighted by a yellow-red color gradient coding for differences in proliferation rates across the fin. (a,c) The preponderance of red at the center of the fin shows where the bulk of cell divisions takes place, with only a few of them occurring near the lateral surfaces (yellow). (b) A decreasing gradient of proliferation rates from the proximal pole to the distal tip characterizes the PD axis. (d-f) Marginal distributions of proliferation along the AP, PD and DV axes respectively, expressed in numbers of cells with respect to the absolute distance in μm along the axis. (g-i) Same distributions with respect to the relative distance on the axis. (j-l) Same distributions expressed in proportions of cells with respect to the absolute distance.

picture(0,0)(-35,0)(1,0)30 (0,-35)(0,1)30 picture picture(0,0)(35,0)(-1,0)30 (0,-35)(0,1)30 picture

Supplementary figure 2

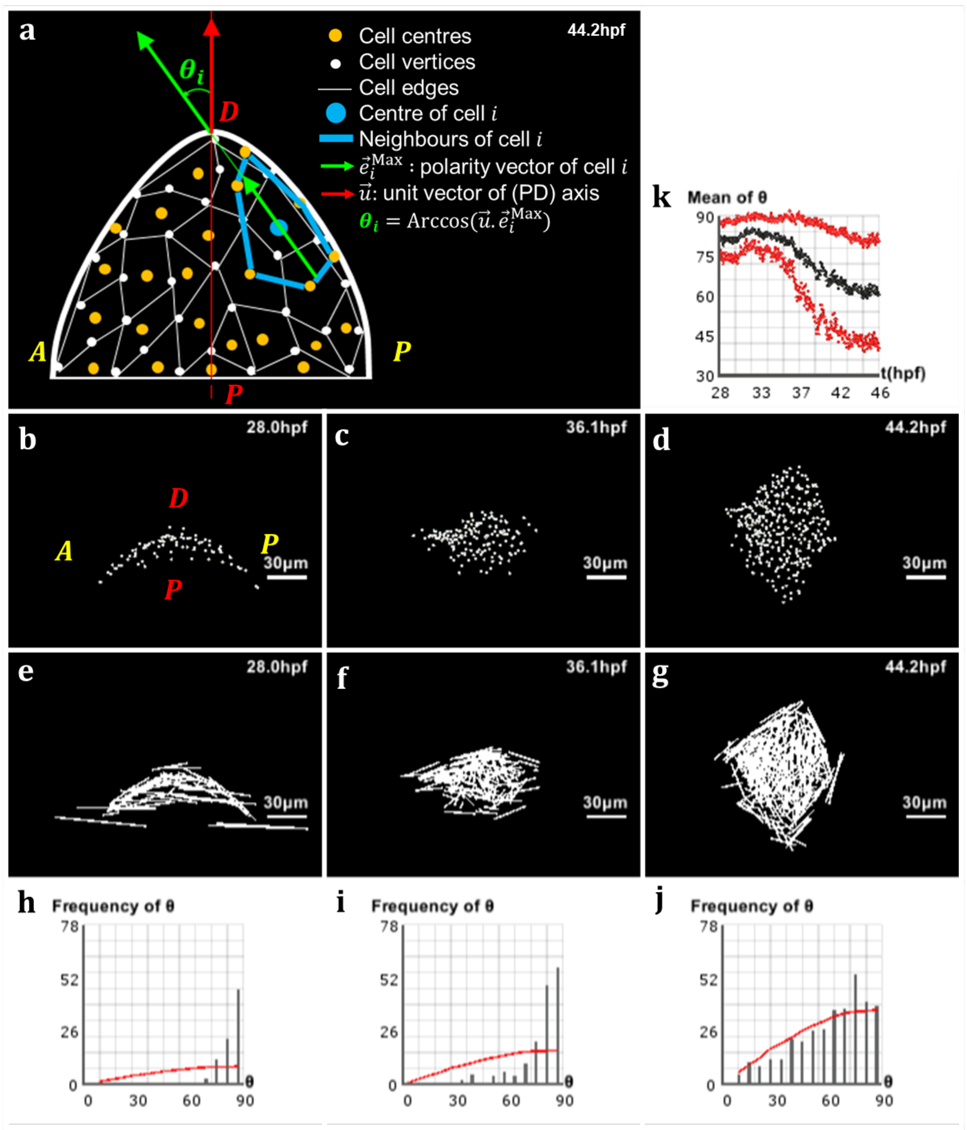

Fig. S2. Analysis of directional cell behaviors in the zebrafish pectoral fin (supplementary dataset). (a) Schematics in 2D of the method used to analyse directional cell behaviors: for each cell i, θ_i denotes the polarity angle that this cell forms between its elongation axis $\overrightarrow{e}_i^{\mathrm{Max}}$ (extracted from the maximum eigenvalue of the covariance matrix of its neighborhood $\mathcal{N}_i$) and the PD axis $\overrightarrow{u}$. (b-d) Lateral view of the pectoral fin at different stages of development, respectively $t = 28.0$ hpf, $t = 36.1$ hpf and $t = 44.2$ hpf. (e-g) Vector field of the cells' elongation axes $\overrightarrow{e}_i^{\mathrm{Max}}$ in the pectoral fin at the same stages. (h-j) Distribution of the polarity angles θ_i of the cells in the pectoral fin at the same stages, compared with the standard distribution of random angles formed by two arbitrary vectors in 3D (red curve). (k) Evolution over time of the average polarity angle $\overline{\theta}$ of the fin cells $\pm$ its standard deviation $\Delta\theta$ shown in red.

picture(0,0)(-35,0)(1,0)30 (0,35)(0,-1)30 picture

picture(0,0)(35,0)(-1,0)30 (0,35)(0,-1)30 picture

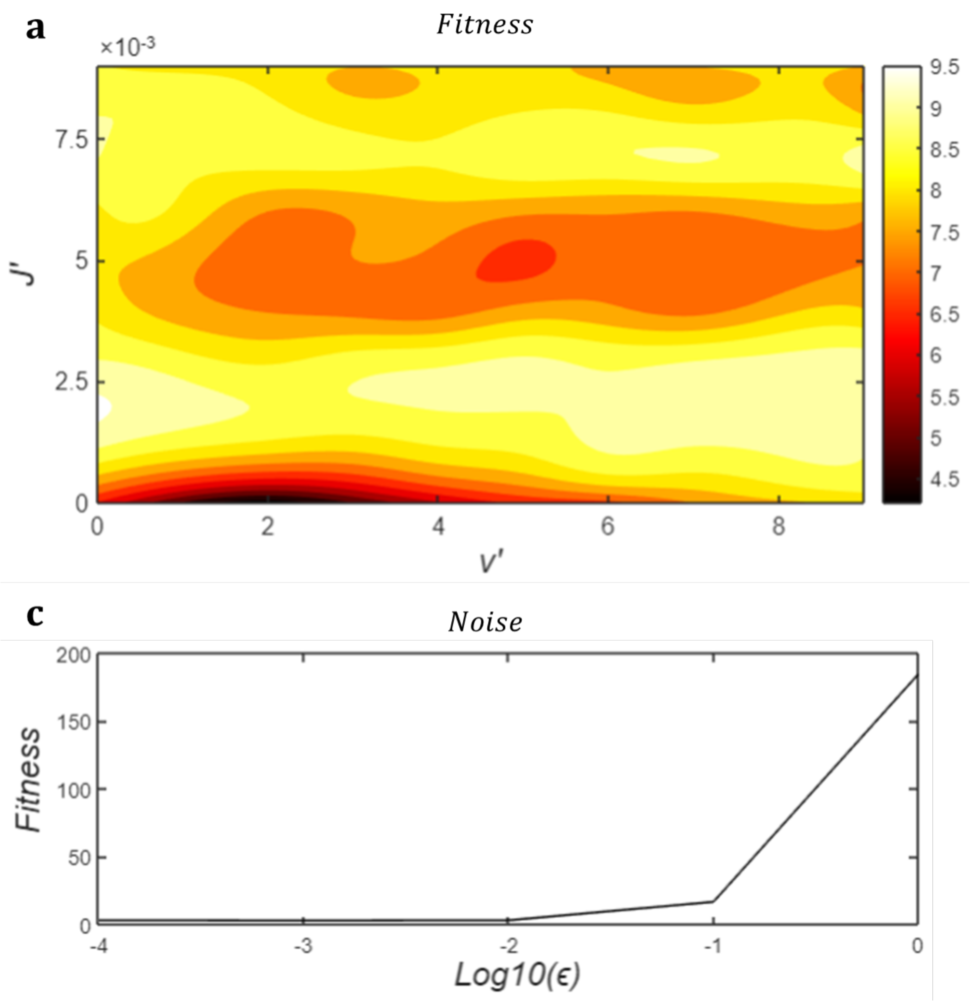

Fig. S3. Analysis of model sensitivity with respect to model parameters and noise. (a) Fitness landscapes as a function of the Attractive-Repulsive force intensity (J') and the polarisation force intensity (ν'). A lower fitness value (red) means a better similarity with the live embryo. (b) Plot of the fitness metric versus noise levels. Here we fixed $\nu' = 8$ and $J' = 0.005$. High levels of noise impact the fitness negatively.

Supplementary figure 3

picture(0,0)(-35,0)(1,0)30 (0,-35)(0,1)30 picture

picture(0,0)(35,0)(-1,0)30 (0,-35)(0,1)30 picture

"output" — 2021/2/1 — page 4 — #4
